# The Paradox of Healthcare in the ‘Superbugs’ Era: Current Challenges and Future Directions

**DOI:** 10.3390/pathogens14121199

**Published:** 2025-11-24

**Authors:** Elenoire Sole, Marilena Trinchera, Silvia De Gaetano, Angelina Midiri, Giovanni Piccolo, Giuseppe Mancuso, Giovanni Schepici, Carmelo Biondo

**Affiliations:** 1 Department of Human Pathology, University of Messina, 98125 Messina, Italy; elenoiresole@icloud.com (E.S.); tmarilena08@gmail.com (M.T.); sdegaetano6@gmail.com (S.D.G.); amidiri@unime.it (A.M.); giovanni.piccolo1@studenti.unime.it (G.P.); mancusog@unime.it (G.M.); 2 IRCCS Centro Neurolesi Bonino Pulejo, 98124 Messina, Italy; giovanni.schepici@studenti.unime.it

**Keywords:** hospital-acquired infections (HAI), superbugs, AMR, MDR

## Abstract

Antibiotic-resistant microbes represent a growing problem for modern medicine and public health. Projections indicate that deaths from such infections could reach 10 million per year by 2050. Healthcare associated infections (HAI) are among the most significant causes of mortality and morbidity in hospitals, impacting millions of patients globally. The emergence of HAI is associated with resistance to antimicrobials, rapidly worsening the patient’s condition. Antimicrobial resistance determines unresponsiveness to treatment, which can ultimately lead to severe complications such as sepsis and shock. It is estimated that one in every ten patients are susceptible to infection during their stay in hospital, with the microorganism responsible for the infection frequently proving resistant to antibiotics. Among the latter, CRE (carbapenem-resistant *Enterobacteriaceae*), CRAB (carbapenem-resistant *Acinetobacter baumannii*), CRPA (carbapenem-resistant *Pseudomonas aeruginosa*), vancomycin-resistant *Enterococcus* spp. and methicillin-resistant *Staphylococcus aureus* (MRSA), commonly referred to as ‘superbugs’, are a major cause of HAIs. The aim of the present study is to provide a comprehensive overview of the global epidemiology of healthcare-associated infections, with particular emphasis on their incidence, distribution over time, and correlation with the socioeconomic status of different countries. Furthermore, the review aims to evaluate the effectiveness of current preventive strategies in reducing the incidence and mortality associated with HAIs.

## 1. Introduction

Every year, millions of patients worldwide are affected by healthcare-associated infections (HAIs), including those caused by microorganisms that no longer respond to drugs like antibiotics [1]. These HAIs pose a serious threat not only to patients and healthcare workers but also, potentially, to family members and visitors to healthcare facilities [2]. These infections have the potential to be contracted at any stage of healthcare delivery and can spread through outbreaks in healthcare settings [3]. It is estimated that the probability of contracting an infection during a stay in an acute care hospital is 15% for patients in low-middle-income countries and 8% for patients in high-income countries [4]. The global spread of microorganisms resistant to antibiotic therapy is a serious public health concern that has multiple causes and requires fundamental changes in the way antibiotics are used [5]. It is estimated that over 39 million people worldwide could potentially be at risk of death from antibiotic-resistant infections over the course of the following twenty-five years [6]. Nevertheless, it is estimated that enhanced access to healthcare and antibiotics could result in the saving of 92 million lives between 2025 and 2050 [6]. The emergence of antibiotic resistance has severe consequences, including increased mortality and healthcare expenditures due to prolonged hospital stays, an increase in hospital admissions, and the need to prescribe more toxic and expensive antibiotics [7,8]. Furthermore, while the phenomenon of antibiotic resistance was initially associated with microorganisms responsible for hospital-acquired infections (i.e., infections developing in hospitalised patients), recent reports also indicate the spread of antibiotic-resistant bacterial strains within the community [9,10]. The phenomenon of mono- and multi-microbial resistance is now widely recognised as a global health risk, impacting humans on a worldwide scale [11]. Risk perception has broadened from hospital-based to general population. There are rising reports of drug-resistant microorganisms, called “superbugs”, causing significant illness and death, especially for immunocompromised people [10,12]. A specific group of virulent, antibiotic-resistant bacteria that cause a significant number of infections, especially in hospitals, is known by the ESKAPE acronym: *Enterococcus faecium*, *Staphylococcus aureus*, *Klebsiella pneumoniae*, *Acinetobacter baumannii*, *Pseudomonas aeruginosa*, and *Enterobacter* species. The WHO Bacterial Priority Pathogens List, published in 2024, is a significant instrument in the global effort to combat antimicrobial resistance (AMR). This edition updates and refines the prioritisation of antibiotic-resistant bacterial pathogens, building on the 2017 WHO BPPL. The objective of this study is to address the evolving challenges of antibiotic resistance. The categorisation of these pathogens into critical-, high- and medium-priority groups is facilitated by the list. The 2024 WHO BPPL encompasses 24 different pathogens, among which are Gram-negative bacteria demonstrating resistance to last-resort antibiotics [10]. Examples of such pathogens include, but are not limited to, *P. aeruginosa*, *Staphylococcus aureus*, *Mycobacterium tuberculosis* and *Neisseria gonorrhoeae*. This document is a guideline for the prioritisation of public–private partnerships for antibiotic R&D and AMR programme development and implementation. Moreover, the list is intended to assist public health officials in determining the most effective interventions, including surveillance and prevention measures, to mitigate the propagation of resistance and to devise strategies for outbreak readiness.

### The Global Issue of Antimicrobial Resistance

The increased demand for antibiotics in industrialised nations can be primarily attributed to the advancement of medical technologies [13]. These technologies have enabled a greater range of complex medical procedures, necessitating the use of antibiotics to prevent infections. The employment of sophisticated technologies, including organ transplants, chemotherapy and complex surgical interventions, invariably gives rise to patient populations that are vulnerable to bacterial infections [14]. Consequently, the employment of antibiotics becomes imperative for the successful and safe execution of these complex medical procedures [15,16]. Effective therapeutic strategies have also led to a greater prevalence of conditions that result in immunosuppression, as seen in individuals suffering from various oncological diseases undergoing intensive chemotherapy [17,18]. The ability to contain the rise in the AMR phenomenon depends on effective antibiotic therapy management. This involves interventions to regulate key aspects, such as antibiotic selection, duration of treatment, dosage appropriateness, and distinction between therapeutic and prophylactic use of antibiotics [19,20]. The AMR phenomenon also requires the establishment or strengthening of surveillance systems at national and international levels [21]. In this context, the implementation of Infection Prevention and Control (IPC) programmes and Water, Sanitation and Hygiene (WASH) services is of paramount importance in order to prevent the transmission of infection [22,23]. This study aims to provide a comprehensive review of the global epidemiology of HAIs, focusing particularly on their incidence, the temporal distribution of these infections, and the association with the socioeconomic status of diverse nations. Furthermore, the efficacy of contemporary preventive strategies in diminishing the occurrence and mortality associated with HAIs will be assessed.

## 2. Most Common Healthcare Infections

**Figure 1:**
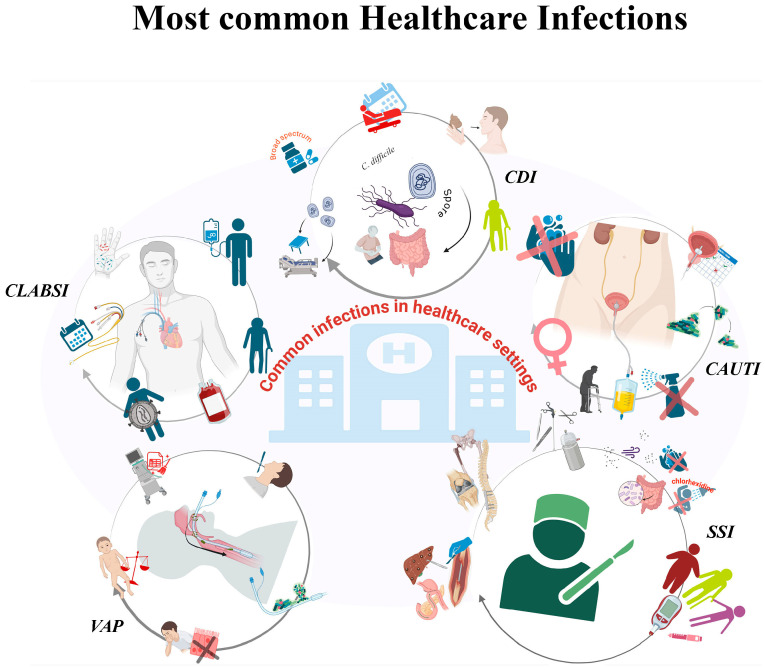
Healthcare-associated infections (HAIs). Figure created using BioRender.

The pathogens responsible for HAIs have been isolated from a variety of sources, including but not limited to patients and the hospital setting [24,25]. The most frequent HAIs are: central line-associated bloodstream infections (CLABSI), catheter-associated urinary tract infections (CAUTI), ventilator-associated pneumonia (VAP), surgical site infections (SSI) and gastrointestinal infections caused by *Clostridioides difficile* [26,27] (Figure 1).

The most prevalent healthcare-associated infections (HAIs) encompass urinary tract infections (UTIs), respiratory tract infections, surgical site infections (SSIs), and central line-associated bloodstream infections (CLABSI), ventilator-associated pneumonia (VAP) and gastrointestinal infections caused by *Clostridioides difficile* (CDI). Risk factors vary, but frequently encompass invasive medical devices, surgical procedures, compromised immune systems and suboptimal hygiene.

### 2.1. Central Line-Associated Bloodstream Infections (CLABSI)

According to the CDC (Centres for Disease Control and Prevention), a central line-associated bloodstream infection is defined as the recovery of a pathogen from a blood culture in a patient who had a central line at the time of infection or within 48 h prior to the onset of infection [28]. This definition involves either a single blood culture for an organism not commonly present on the skin or two or more blood cultures for organisms commonly present on the skin. According to the IPC Global Report 2024, CLABSIs account for 13% of HAIs [29]. Among all HAIs, CLABSI has been found to be associated with a high mortality rate, with attributable mortality ranging from 12% to 25% [30,31]. There are two types of central venous catheters (CVC): tunnelled and non-tunnelled. Tunnelled catheters are designed for long-term use and are put under the skin [32]. Non-tunnelled catheters are put into a vein. These are at higher risk of CLABSI because they don’t have a subcutaneous tract and are used for shorter periods [32,33,34]. A number of risk factors have been identified in relation to the development of CLABSI, with one of the most significant being the utilisation of a central line over an extended duration. The odds ratio for this range is between 1.028 and 5.52 [35]. The fundamental rationale underlying this phenomenon pertains to the enhanced capacity of bacteria to adhere to the catheter, thereby facilitating the onset of an infection [36]. Individuals with compromised immune systems, including those undergoing chemotherapy, transplant recipients, or those living with HIV, are at an increased risk of developing CLABSI [35]. A number of factors may be implicated in the causation of this condition, including but not limited to: inadequate hand hygiene, improper insertion of the catheter, and the utilisation of catheters devoid of a coating [37]. The latter have a special protective layer, making them safer and easier to use. The microrganisms most commonly associated with CLABSI are those found on the skin or in the environment, including *Enterobacteriaceae* (family) (23–31%), *Staphylococcus* (genera) (16%), *Candida* (genera) (27.6%), coagulase-negative staphylococci, *Enterococcus* (genera) (16%) and *Pseudomonas* (genera) (3.1%) [35,38]. When patients exhibit signs of CLABSI, empirical therapy is recommended to ensure adequate coverage for common Gram-positive and Gram-negative organisms [39]. Local data and antibiograms must be considered when awaiting culture and sensitivity results as treatment varies depending on the prevalence of resistant organisms and other factors [40]. For instance, in cases of prevalent MRSA, start with parenteral vancomycin [39]. When there is a high risk of drug-resistant bacteria, a combination of a beta-lactam (ceftazidime or cefepime), a lactamase inhibitor, and an aminoglycoside is indicated. Conversely, if there is a likelihood of candidemia and azole resistance, the use of echinocandins is recommended [28,41]. Following the availability of the results of the aforementioned susceptibility tests, the initiation of appropriate therapy must be considered. In the event that blood cultures demonstrate no growth, despite the patient continuing to exhibit fever, it is imperative to remove the catheter and submit the tip for culture analysis [42]. While the prompt removal of all non-tunnelled catheters is imperative, the utilisation of long-term catheters with “salvage therapy” is a viable alternative when removal is either unfeasible or undesirable [43]. The objective of this approach is to eradicate infection and preserve the catheter, particularly in patients with limited access or undergoing long-term therapies such as haemodialysis [44]. CLABSI is a prevalent problem in ICUs, leading to many deaths and financial burdens. The only viable strategy for achieving a complete elimination of CLABSI necessitates a multifaceted approach, encompassing stringent adherence to optimal practices, established protocols and checklists [45]. The latter is a system that provides healthcare personnel with step-by-step guidance through critical procedures, ensuring the correct performance of each essential action and minimising human error. The CDC and the IDSA have published a comprehensive prevention programme, which includes:

(a) Maintaining good hand hygiene by washing hands with soap, water, or alcohol-based gels or foams. Gloves do not prevent the need for hand hygiene.
(b) Employing rigorous aseptic techniques and sterile barrier precautions.
(c) Disinfecting the skin with 2% chlorhexidine before inserting central venous catheters.
(d) Using ultrasound guidance to optimise placement, minimise mechanical complications, and reduce repeated attempts.
(e) Avoid the femoral vein for central line placement, using the subclavian vein when possible for non-tunnelled catheters.
(f) Removing any central line when it is no longer required.
(g) Meticulously disinfecting the catheter hubs, injection ports and connections prior to accessing the line.

The utilisation of a checklist is recommended, as this is a tool used in planning a programme designed to prevent a specific risk [35,46].

### 2.2. Catheter-Associated Urinary Tract Infections (CAUTI)

Urinary tract infections (UTIs) are a prevalent cause of sepsis in hospitalised patients [47]. Despite the existence of a variety of classification schemes, the CDC’s are the most widely utilised. The latter distinguishes between uncomplicated and complicated UTIs based on abnormalities in the urinary tract or other conditions that increase the risk of treatment failure [48]. While bacteriuria is generally not considered to be a UTI in the absence of symptoms and therefore does not necessitate treatment, a non-complicated UTI (i.e., cystitis) is defined as a localised urinary tract infection, devoid of any indications of systemic infection, attributable to susceptible pathogens and not associated with treatment failure or unfavourable outcomes [48,49]. A UTI is deemed complicated if it does not conform to the aforementioned description and may be associated with fever, renal calculi, sepsis, urinary obstruction, or the use of a catheter. Complicated UTIs are also associated with pregnancy, diabetes, neurogenic bladder, renal disease, and other immunocompromising conditions, and are linked to a higher risk of treatment failure and reinfection [50]. Around 75% of all hospital-acquired UTIs are catheter-associated (CAUTIs), and the risk of contracting these infections increases with prolonged use of a urinary catheter [51]. Such conditions have the potential to result in the onset of urosepsis, with a mortality rate of 30% [52]. The IDSA and the CDC define catheter-associated urinary tract infections (CAUTIs) differently [53]. The CDC focuses exclusively on the management of symptomatic cases, whereas the IDSA encompasses both symptomatic and asymptomatic cases [51]. The CDC relies heavily on urine culture results, while the IDSA also considers clinical signs and symptoms. It is estimated that up to 25% of all patients receiving hospital care will have a urinary catheter at some point during their hospitalisation [54]. Furthermore, research has indicated that 20% of hospital-acquired bacteraemia in acute care facilities were attributable to urinary catheterisation, underscoring the significance of these types of catheters as a potential source of bloodstream infections in such facilities [55,56]. It is estimated that up to 50% of such catheters are unnecessary and non-compliant, resulting in thousands of deaths and hundreds of thousands of infections per year [57]. Catheter patients are expected to have bacteriuria due to bacterial colonisation and the development of biofilms. The rate of bacterial colonisation is high and increases by up to 100% after 30 days [58]. The primary risk factors that have been identified include being female, age, diabetes, and errors in catheter insertion and maintenance care [53]. Collecting urine specimens from catheterised patients is vital in cases suspected of CAUTI. The primary objective is to avoid culturing the biofilm [59]. Numerous studies have demonstrated a correlation between the insertion of the catheter and the onset of a chronic inflammatory state, characterised by the accumulation and deposition of fibrin. This substrate is well-suited to the production of biofilm. For instance, it has been determined that *Enterococcus faecalis* possesses the capacity to synthesise and secrete a protease, designated as SprE, which has been shown to enhance the accumulation of fibrin. Consequently, this results in the generation of an environment conducive to the colonisation and production of biofilm. Furthermore, this has the potential to elevate the likelihood of the development and dissemination of polymicrobial infections at a systemic level [60]. The guideline recommends that, where practicable, the catheter should be removed and the patient asked to urinate for the purpose of specimen collection. If this isn’t possible, it is recommended that the catheter be replaced prior to specimen collection. Never collect urine cultures from the drainage bag [61]. The most frequent organisms causing CAUTIs are *E. coli* (24%), *Enterococcus* (14%), *Pseudomonas* (10%), and *Klebsiella* (10%). Many organisms are increasingly resistant to a range of antibiotics, including fluoroquinolones, cephalosporins, aminoglycosides, and carbapenems. Yeast species belonging to different *Candida* genera have also increased (24%) [62].

### 2.3. Ventilator-Associated Pneumonia (VAP)

Ventilator-associated pneumonia (VAP) is a lung infection that develops in patients on mechanical ventilation for over 48 h. It is estimated that between 5–40% of patients treated with invasive mechanical ventilation for more than 2 days are affected by VAP, with a mortality rate of approximately 10%. VAP is the second most prevalent hospital-acquired infection among paediatric intensive care unit patients, with prematurity and long ventilation being the key risk factors. The clinical signs presented included fever, respiratory distress and purulent tracheal discharge [63,64]. This condition is characterised by a bacterial infection, typically caused by a single organism, usually *Staphylococcus aureus* (28.4%) or *P. aeruginosa* (25.2%) [65]. Previous studies have indicated an observed rise in polymicrobial infections, with no discernible distinction observed between adult and paediatric patients in ICU settings [66]. In addition to the aforementioned pathogens, *Klebsiella*, *Enterococcus*, *Streptococcus* and *Acinetobacter* species have been identified as capable of colonising artificial airways following intubation or tracheostomy, a phenomenon that has been particularly prevalent in patients receiving care in the Neonatal Intensive Care Unit [67]. Furthermore, while viruses and fungi are rare causative agents of VAP, anaerobic bacteria have been identified as a cause of pneumonia, particularly when it is due to aspiration [65,68]. The primary mechanism underlying the development of VAP is the progression of upper airway colonisation, which subsequently leads to tracheal colonisation, tracheitis, and ultimately, pneumonia [69]. The likelihood of a host contracting an infection is determined by two main factors: the virulence of the pathogen and the host’s intrinsic defences, which can be categorised as mechanical, humoral or cellular immunity [70]. However, these can be altered in an intubated patient, since the presence of an artificial airway inhibits the gag reflex and ciliary functions. Furthermore, these artificial airways act as a substrate for the growth of a biofilm that can be detached and delivered to the lower respiratory tract through mechanical aspiration resulting in pneumonia [71,72]. The diagnosis of VAP remains a significant challenge in the clinical setting, even in cases where positive culture samples are obtained from intubated patients. This is due to the fact that the majority of these patients are colonised within 48 h, and the clinical value of such a sample is limited [73]. The duration of intubation is directly correlated with the increased probability of developing VAP [74]. Quantitative bacterial cultures from the lower respiratory tract are often obtained via bronchoscopic bronchoalveolar lavage (BAL) in adults, but this method is impractical for children and impossible for newborns due to the risk of complications [75]. Thus, only clinical criteria are used to diagnose ventilator-associated pneumonia in infants under one [76]. In cases where the presence of VAP is suspected, the initiation of broad-spectrum therapy is recommended. The latter should be founded upon two factors: firstly, the severity of the clinical disease, and secondly, the knowledge of local pathogen susceptibility patterns. Broad-spectrum therapy should include coverage of Gram-negative bacilli, including *P. aeruginosa*, and possibly methicillin-resistant *S. aureus* [63,65]. Main risk factors for ventilator-associated pneumonia include: (1) treatment that promotes colonisation of the oropharynx or stomach; (2) factors that promote gastric reflux and aspiration; (3) duration of mechanical ventilation; (4) advancing age and comorbidity [65,77]. In order to reduce the incidence of ventilator-associated pneumonia, a variety of strategies may be employed, including the appropriate use of reflux medication, the removal of unnecessary nasogastric tubes and the maintenance of mechanical ventilation systems [78].

### 2.4. Surgical Site Infections (SSI)

Surgical site infections (SSI) are considered the most significant source of hospital-accquired infections among surgical patients [79]. These infections contribute to 20% of all HAIs [79]. Postoperative wound infections are common after surgery and have complex causes. In the context of an operation, the maintenance of sterility and cleanliness within the operating room environment is of paramount importance, given its direct impact on infection rates [80]. The symptoms associated with SSI include redness, warmth, pain and swelling, which can be similar to the symptoms associated with cellulitis or allergies. Signs of infection that may be important for diagnosis include pus, drainage and fever [80,81]. Diagnosis is based on a medical exam, but samples may need to be cultured and imaged to confirm the diagnosis. Recent data demonstrate that SSI are responsible for a significant number of postoperative complications and mortalities, accounting for over 2 million infections in the U.S. [80]. The CDC categorises SSI into the following categories: superficial, deep incisional, or organ/space infections. Such infections must occur within 30 days of surgery (or within one year if an implant is involved) for an SSI to be diagnosed [82]. The majority of SSI are caused by pathogens found on mucous membranes, skin or hollow viscera. Superficial incisional infections, affecting the skin and subcutaneous tissues, account for over 50% of all surgical site infections [83]. However, the aetiology of SSI is multifactorial, encompassing direct contact, airborne transmission and endogenous microbe contamination. The following factors are considered to be the primary risks of SSI for patients: advanced age; malnutrition; obesity; steroid use; diabetes; an immunocompromised state; trauma; and the location of the procedure site (intraabdominal, pelvic, or extremity) [79,84]. Extended preoperative hospitalisation and inadequate preoperative skin hygiene have also been identified as significant risk factors. Adherence to the surgical safety checklist is of paramount importance in order to achieve a reduction in the occurrence of surgical site infections and mortality rates [85]. Optimal ventilation is crucial and can be achieved through positive pressurisation. Reducing patient skin microbiota with a preoperative chlorhexidine shower is recommended, either the night before or on the day of surgery. Hair should be removed using clippers immediately before surgery (except scrotal surgery) [86]. According to the most recent estimates, the prevalence of SSI among surgical patients is approximately 3%. However, the available data underestimates the growing prevalence of outpatient surgery [87]. Despite advanced preventive measures, surgical site infections are a significant challenge, affecting patient morbidity and mortality. These infections lead to higher ICU admission rates and a two to eleven times higher mortality risk, as well as a five times greater probability of further hospitalisation [79,88]. Moreover, the estimated mean duration of hospitalisation due to surgical site infection has been determined to be 10–11 days, with an additional financial burden exceeding $20,000 per admission [89]. SSI can be caused by either endogenous or exogenous agents. The presence of endogenous microbes can be attributed to various sources, including the patient’s skin, mucous membranes, or intestines, with the potential for dissemination via the blood. The most common endogenous causative organisms of SSI vary depending on the anatomical site [90]. For instance, in the post-operative phase of breast, orthopaedic, cardiac and vascular surgeries, *S. aureus* and coagulase-negative staphylococci are frequently implicated. Conversely, following surgical interventions on the peritoneal cavity, an increased prevalence of *Enterococcus*, Gram-negative bacilli, and anaerobes as causative agents has been reported [91,92]. Exogenous microbes may originate from the operating room environment and be transmitted through the air or via hospital staff. Frequently identified organisms in surgical site infections include staphylococci and streptococci [93]. However, the prevalence of highly virulent hospital-acquired pathogens, such as methicillin-resistant *S. aureus* (MRSA) or extended-spectrum β-lactamase Gram-negative bacteria isolated from surgical site infections, is increasing. For instance, in the United States, the incidence of MRSA-associated surgical site infections has been observed to steadily increase, having nearly doubled from 23% in 2005 to 43.7% in 2010 [94]. Open wounds and drainage, especially if purulent, require cultures to select the correct antibiotics. The presence of standard negative cultures may be indicative of unusual infections, such as mycobacteria or yeasts. In such cases, it is essential to obtain specific cultures for these microrganisms to ensure appropriate treatment [95].

### 2.5. Gastrointestinal Infections Caused by Clostridioides difficile

*Clostridioides difficile*, formerly known as *Clostridium difficile*, is a Gram-positive and spore-forming bacterium that has been identified as the primary cause of healthcare-associated colitis [96]. It poses a considerable public health challenge, primarily due to the transmissibility of the infection and the subsequent risk of morbidity and mortality. This obligate anaerobic bacillus is considered a primary HAIs, commonly transmitted via contaminated surfaces in hospitals, often in the form of spores. However, there has also been an observed rise in community-acquired *C. difficile* infections [97]. Symptoms of *C. difficile* infections vary widely and range from being asymptomatic (where the bacterium is present in the gut without symptoms) to diarrhoea. In severe cases, there may be a progression of the infection to pseudomembranous colitis and toxic megacolon with subsequent septic shock and high mortality rates [98]. *C. difficile* transmission is primarily via faecal-oral route, but also via contaminated healthcare facilities. Spores can be found on surfaces where they can function as reservoirs for this bacterium, with the possibility of transmission if cleaning practices are inadequate [99]. Numerous studies suggest that the primary risk factor for *C. difficile* infection is the use of broad-spectrum antibiotics [97,100]. The administration of various antibiotics, including cephalosporins and fluoroquinolones, has been demonstrated to exert a disruptive influence on the equilibrium of the gut microbiome (i.e., patients may be carriers of *C. difficile* without exhibiting symptoms), thereby giving rise to dysbiosis and consequently facilitating the proliferation and infection of *C. difficile* [100,101]. A number of additional risk factors have been identified, including older age, pre-existing comorbidities, impaired immune response, and prolonged hospitalisation. This Gram-positive bacterium produces two toxins, A and B, encoded by tcdA and tcdB genes. These toxins damage host cells and disrupt the colon’s epithelial barrier [102]. Most pathogenic *C. difficile* strains produce both toxins, with toxin B being characterised as more potent. Furthermore, it has been observed that certain strains of *C. difficile* are capable of producing solely toxin B. Moreover, some *C. difficile* strains also produce a third toxin, binary toxin CDT, which enhances inflammation, contributes to disease severity and fluoroquinolone resistance [103,104]. The healthcare environment has been identified as a setting for *C. difficile* transmission, largely due to the misuse of antibiotics. It has been estimated that between 30% and 50% of prescribed antibiotics in hospitals are either unnecessary or inappropriate, thereby increasing the risk of *C. difficile* infections [105]. Recent data from the United States and Europe demonstrate a decrease in the prevalence of *C. difficile* in healthcare systems. This is attributable to a range of measures, including the implementation of antibiotic stewardship practices and the enhancement of infection control procedures [106].

## 3. Why Do Hospital-Acquired Infections Occur?

Hospital-acquired infections, also called ‘healthcare-associated infections’ are defined as such if symptoms appear 48 h after admission to hospital, within three days of discharge or 30 days after surgery [107]. These infections have the potential to be acquired in a variety of healthcare settings, including intensive care units, clinics, surgical centres, nursing homes, care facilities, dialysis centres and laboratories. Hundreds of millions of patients worldwide are affected by preventable infections contracted during healthcare delivery [24]. Consequently, it is crucial to understand the gaps in hospital-acquired infection control. HAIs are often linked to inadequate water, sanitation, and safety programmes, as well as technologically advanced and invasive health treatments [6]. The Global Report on IPC 2024 states that 9% of countries have not implemented an IPC programme. The third of the study’s participants had IPC implemented at national level, with functional health facilities, including sanitation and water, as well as the necessary equipment for control and prevention. However, only 6% of these participants had met all the minimum requirements requested by the WHO [26]. However, the prevalence of HAIs may vary based on socioeconomic conditions, local politics, and observation methodology. A review of the literature on HAIs in the United States underscored the pivotal role of social determinants of health. The highest rates of central venous and urinary catheter-associated infections were observed among patients from ethnic groups experiencing elevated levels of social vulnerability, including Hispanics, Asians, and Blacks [108]. It is estimated that 1 in 10 patients experience adverse health consequences as a result of substandard healthcare, with a global mortality toll of over 3 million [109]. In low- to middle-income countries, as many as 4 in 100 deaths are attributable to unsafe care. In such contexts, risk factors (e.g., water shortage, poor hygiene, staff shortages) are compounded by other determinants (e.g., overcrowding, malnutrition, low birth weight). This is especially evident in rural sub-Saharan Africa (59% of the population resides there), as opposed to urban areas [110]. A study in a Malawian hospital highlighted poor hand hygiene practices by health personnel. Moreover, the limited availability of resources has a direct impact on the number of diagnostic facilities available. The absence of imaging supports, such as MRI and CT scans, and microbiological laboratories, significantly restricts the capacity for effective infection prevention and control [111]. Adverse events are estimated to be preventable 50% of the time, with medication contributing half of these events. In addition, 40% of patients suffer harm in primary and ambulatory care [112]. The preponderance of hospital-acquired infections is attributable to bacterial pathogens, with the dissemination of these infections being exacerbated by the proliferation of multidrug-resistant bacterial strains [113]. According to recent data from the CDC, of all patients staying in an ICU for a period longer than two days, 10% presented with pneumonia, 8% with bloodstream infection and 4% with urinary tract infection [114,115]. Immunocompromised individuals, such as chemo patients and the elderly with multiple comorbidities, are more susceptible to hospital-acquired infection. The longer a patient spends in hospital, the higher the risk. The same is true of those receiving mechanical ventilation, undergoing surgery, or with indwelling devices [116]. A large-scale study conducted across 947 European hospitals between 2015 and 2016, involving 231,459 patients, found that 19.5% of ICU patients contracted at least one hospital-acquired infection [117]. The repercussions of patient harm also have a detrimental effect on economic resources. According to estimates by the Organisation for Economic Co-operation and Development (OECD), adverse events constitute 15% of hospital expenditures and services. Adverse events have the capacity to affect patients at all stages of their care [112]. While priorities are subject to variation in accordance with the specific characteristics of each nation and its respective healthcare system, the primary issues pertaining to patient safety are consistent, as identified by the WHO [118]. Table 1 provides a comprehensive overview of the most prevalent medical errors and the associated complications.

## 4. Tackling the Superbug Challenge

**Figure 2:**
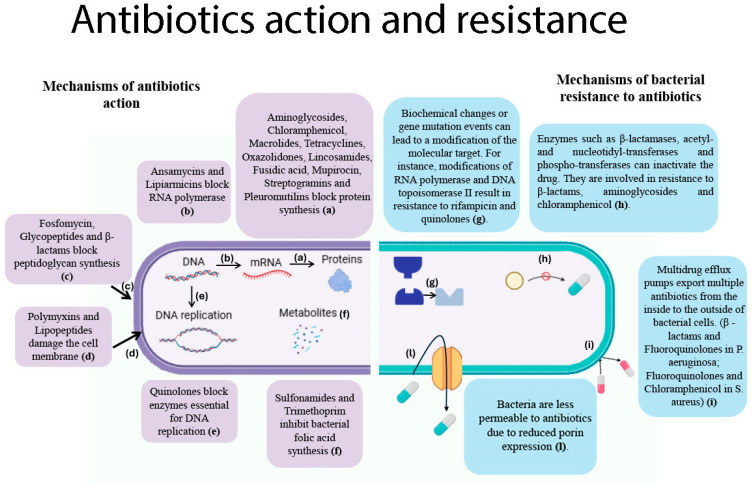
Action mechanisms and resistance in commonly used antimicrobials. (**Left panel**): Mechanisms of action of antibiotics. (**a**), Aminoglycosides, chloramphenicol, macrolides, tetracyclines, oxazolidinones, lincosamides, fusidic acid, mupirocin, streptogramins and pleuromutilins inhibit protein synthesis by acting on ribosomal activity; (**b**), Ansamycins block the action of RNA polymerase, stopping DNA-to-mRNA transcription; (**c**), Fosfomycin exerts its inhibitory effect on the initial step in the biosynthesis of peptidoglycan, whereas glycopeptides and β-lactams are effective at the late stage of this process; (**d**), Polymyxins and lipopeptide antibiotics damage bacteria in different ways. Polymyxins disrupt Gram-negative outer membranes. Daptomycin, a common lipopeptide, forms aggregates in the membrane, causing leakage and possibly inhibiting cell-wall synthesis; (**e**), Quinolones trap bacterial enzymes on DNA, forming drug–enzyme–DNA complexes. This process blocks the DNA replication fork and causes DNA breaks, thereby inhibiting bacterial DNA replication; (**f**), Sulfonamides and trimethoprim inhibit bacterial folic acid synthesis at different stages. Sulfonamides mimic para-aminobenzoic acid (PABA), preventing the formation of dihydrofolic acid. Trimethoprim blocks the reduction of dihydrofolic acid to tetrahydrofolic acid, stopping dihydrofolate reductase. (**Right panel**): Mechanisms of bacterial resistance to antibiotics. (**g**), Spontaneous genetic mutations in RNA polymerase and DNA topoisomerase II can cause antibiotic resistance during bacterial replication, reducing the binding of the antibiotics rifampicin and quinolones to their target enzymes. Resistant strains emerge and spread; (**h**), Gram-negative bacteria have the capacity to produce enzymes such as β-lactamases and aminoglycoside-modifying enzymes that have the capacity to degrade or modify antibiotics. These microorganisms demonstrate resistance to β-lactams, aminoglycosides and chloramphenicol; (**i**), Multidrug efflux pumps facilitate the export of antibiotics from the cell, thereby contributing to the development of resistance. Examples of such systems include the MexAB-OprM system found in *Pseudomonas aeruginosa*, which confers resistance to beta-lactams and fluoroquinolones, and the NorA pump found in *Staphylococcus aureus*, which confers resistance to fluoroquinolones and chloramphenicol; (**l**), Decreased porin expression (e.g., the loss of OmpD porin in *Pseudomonas aeruginosa* and *Escherichia coli*) has been demonstrated to reduce the channels available for antibiotics to enter the cell, thereby increasing resistance to carbapenems. Figure created using BioRender.

Since their introduction into clinical practice, antibiotics have played a vital role in healthcare, saving lives and improving patient outcomes [119]. Furthermore, they have played a pivotal role in the success of high-risk clinical procedures, including surgeries involving infection, organ transplants and other invasive interventions. Nevertheless, there has been a growing increase in the phenomenon of antibiotic resistance in recent decades [9]. This natural phenomenon enables bacteria to defend themselves against these compounds; however, it has been significantly accelerated by the misuse and overuse of antibiotics. The phenomenon of AMR in bacteria is the result of a number of key mechanisms, which are outlined in Figure 2.

These mechanisms include alterations to drug targets, active efflux, reduced membrane permeability, and enzymatic inactivation of antimicrobial agents [9,10]. Within the context of the clinical setting, AMR has emerged as a significant challenge, particularly with regard to certain bacterial pathogens. These pathogens have developed resistance to antimicrobial treatments, including “last resort” antibiotics, leading to the emergence of so-called antimicrobial-resistant superbugs [12]. The following pathogens are of particular concern: vancomycin-resistant enterococci (VRE) among Gram-positive cocci, and carbapenem-resistant *Enterobacterales* (CRE), carbapenem-resistant *A. baumannii* (CRAB), and extensively drug-resistant *P. aeruginosa* (XDR-PA) among Gram-negative bacilli.

### 4.1. Methicillin-Resistant Staphylococcus aureus (MRSA)

MRSA has now become a significant causative agent of both hospital-acquired and community-acquired infections [120]. This phenomenon can be attributed to the acquisition of the *mecA* gene by this Gram-positive bacterium, which encodes a penicillin-binding protein 2a (PBP2a) capable of facilitating cell wall synthesis while exhibiting a low affinity for beta-lactam antibiotics such as methicillin The ability of bacteria to continue producing cell walls and replicating even in the presence of antibiotics, thereby rendering methicillin ineffective, complicates the management of pneumonia, sepsis and osteomyelitis [121]. MRSA strains contain a mobile genetic element designated SCCmec (staphylococcal cassette chromosome), which carries the *mecA* gene, thereby conferring resistance to beta-lactam antibiotics. These SCCmec elements have also been observed to incorporate additional genes, which can confer resistance to other antibiotic classes, including macrolides, lincosamides, aminoglycosides, streptogramins B, and tetracyclines. This contributes to the development of multidrug-resistant (MDR) strains [122]. Moreover, intergeneric exchanges have also been observed. These are gene cassettes originally present in Gram-negative bacteria, consisting of genes for resistance to streptomycin, chloramphenicol and trimethoprim, which were subsequently also described in Gram-positive bacteria. The occurrence of these exchanges is of concern, as it significantly increases the spread of antibiotic resistance [123,124].

### 4.2. Multidrug-Resistant Gram-Negative Bacterial Infections

The 2023 annual epidemiological report by the European Centre for Disease Prevention and Control (ECDC) has confirmed a 57.5% increase in carbapenem-resistant *K. pneumoniae* (CRKP) bloodstream infections (BSIs) in the European Union (EU), with an incidence of 3.97 cases per 100,000 inhabitants compared to 2.52 cases in 2019. This increase indicates that the objective of reducing CRKP infections by 2030 will not be achieved, which poses a significant threat due to the high mortality rate associated with such infections [123]. *A. baumannii*, an opportunistic human pathogen, is a significant causative agent of hospital-acquired infections. It has been responsible for approximately 20% of infections associated with intensive care units (ICUs) on a worldwide scale. *A. baumannii* infections predominantly affect ICU patients in critical condition, accounting for nearly 20% of infections in these units [125]. In 2017, the World Health Organization (WHO) included *A. baumannii* on a list of bacteria that were deemed to be critical for new antibiotic research, due to its multidrug-resistant (MDR) phenotype. A significant factor contributing to the resilience demonstrated by this microorganism in hospital settings is their ability to survive in harsh environments. These environments may include conditions of extreme dryness and high salinity, which would typically be lethal to most microorganisms. When disinfection protocols are not adequately followed, this pathogen has been known to persist on hospital surfaces for several weeks. It uses various strategies, including the production or absorption of compatible solutes to protect it from dehydration. One such compatible solute is a sugar alcohol called mannitol, which is better known as a low-calorie sweetener [126]. *P. aeruginosa* is a ubiquitous pathogen that is intrinsically resistant to various classes of antibiotics. It is a principal cause of hospital-acquired infections, which are associated with significant morbidity and mortality [127]. This pathogen has been designated by the WHO as a high-priority pathogen, underscoring the necessity for research and development of novel antibiotics. The emergence of extensively drug-resistant (XDR) *P. aeruginosa* can be attributed to three key factors. Firstly, the extraordinary capacity of this pathogen to develop AMR through chromosomal mutations. Secondly, the increasing prevalence of transferable resistance determinants (i.e., carbapenemases and extended-spectrum β-lactamases). Thirdly, the global dissemination of XDR/Drug-Treatment-Resistant (DTR) high-risk lineages [128]. The microorganism survives on dry surfaces in hospitals for long periods, often contaminating equipment and causing infections. It is the fourth most common hospital-acquired pathogen, accounting for 10% of all HAI. This is due to its ability to form biofilms on surfaces and equipment, and its resistance to commonly used disinfectants [129].

### 4.3. Challenges in Managing ICU Infections

The management of such infections poses a significant challenge, particularly within critical care units. This is due to the limited therapeutic options available for such infections, compounded by the propensity of these conditions to facilitate the dissemination of multidrug-resistant pathogens, which pose a grave threat to patients with severe illnesses [124]. In recent decades, the global dissemination of AMR among various bacterial pathogens has been attributed to the overuse and misuse of existing antimicrobials. Repeated antibiotic treatments can lead to bacterial populations adapting and becoming tolerant and resistant to the drug. This phenomenon of drug tolerance evolves rapidly in response to frequent, intermittent antibiotic treatments. This emerging drug tolerance is specific to the treatment modality. Additionally, it has been shown that the process of tolerance often leads to the development of resistance [130]. The ability of pathogens to produce biofilms on various surfaces contributes to AMR. Biofilms are composed of an extracellular polymeric matrix with DNA, proteins, and polysaccharides. This acts as a barrier, limiting antibiotic penetration and facilitating bacterial escape from the immune system. The microenvironment facilitates gene exchange and the establishment of subpopulations of persistent bacteria, which can evade antibiotics by slowing growth and activity [131]. It is estimated that 60–70% of chronic hospital-acquired infections are caused by biofilms present on the surfaces of medical devices, including pacemakers, mechanical heart valves, vascular grafts, and breast implants [131]. It is evident that the primary factor contributing to the ineffectiveness of the pharmaceutical agent is the inability to achieve the optimal concentration of the drug at the site of infection. The ability of antibiotics to reach their target site is impeded by a combination of reduced porin channels and efflux pumps. A reduction in the uptake and expulsion of the antibiotic results in a lower concentration of the antibiotic inside the bacterial cell, thus contributing to survival and multidrug resistance [132]. The limited target sites caused by different antibiotic molecules are accelerating bacterial resistance (Figure 2). One potential solution to this issue may be to target new biosynthetic pathways with bactericidal effects and to bypass resistance. Furthermore, the dearth of novel drug development has been a contributing factor to the exacerbation of AMR, bearing severe consequences on mortality rates and healthcare expenditure [133]. It has been asserted that this phenomenon may result in a return to a “pre-antibiotic era”, during which common illnesses may once again be fatal [133]. According to the Global Research on Antimicrobial Resistance (GRAM) Project, the estimated direct mortality rate from drug-resistant bacterial infections in 2019 was approximately 1.27 million, representing a substantial rise from the estimated 700,000 deaths recorded in 2016 [133,134]. These findings, derived from statistical models of 23 pathogens and 88 pathogen-drug combinations across 204 countries and territories, underscore the emergence of AMR as a growing global health concern, with projections indicating a further escalation to approximately 10 million deaths per year by 2050, if insufficient action is taken to control AMR [6,135]. The term ‘silent pandemic’ has become a frequently used expression to describe the phenomenon of AMR, emphasising the urgency of addressing it [136]. It is estimated that this issue will result in the deaths of over 39 million people over the next 25 years [10]. As stated in the 2023 publication by the World Health Organisation (WHO) entitled “Antibacterial agents in clinical and preclinical development”, a total of 97 molecules, encompassing 57 traditional and 40 non-traditional antibiotics, are currently under development as candidate antimicrobial agents. Of these, 32 traditional molecules (in Phases 1–3) have been shown to be active against the WHO-designated priority pathogens, particularly CRAB and CRE. However, only 12 of these traditional agents targeting priority pathogens can be considered innovative. The majority of these novel formulations consist of combinations of beta-lactams/inhibitors, antimicrobial peptides and macrolides/ketolides [124]. Recent studies have demonstrated the efficacy of novel beta-lactamase inhibitors, including zidebactam and nacubactam, which have also been shown to be active against class B beta-lactamases (MBL). Furthermore, the molecule BWC0977, a pioneering compound, has been shown to exhibit broad-spectrum antibiotic activity against CRE, CRPA, and CRAB isolates, with a mechanism of action that involves targeting topoisomerase II [137].

### 4.4. Innovative Approaches to Combating Antimicrobial Resistance

A variety of innovative methodologies have been proposed in an attempt to combat AMR. The most promising are outlined below:

(A) Antimicrobial peptides (AMPs) have been shown to target bacterial cell membranes, often resulting in the formation of pores that disrupt cell integrity. Their surfaces, charged in a positive way, attract the bacterial membranes, which are negative in charge. This leads to cell death, e.g., via pore formation causing leakage. AMPs work in a unique way to fight microbes, making it less likely that bacteria will develop resistance to them [47,138].
(B) Metallopolymers: The combination of metallopolymers with traditional antibiotics has been shown to form a new class of antibiotics that are effective against MDR bacteria [139]. Metallopolymers enhance the effectiveness of conventional antibiotics in various ways, including:
  1. Disrupting the bacterial cell membrane, allowing antibiotics to more effectively target and destroy bacteria.
  2. Protecting against resistance mechanisms, e.g., inactivating enzymes that breakdown antibiotics or inhibiting pumps that expel them.
  3. Forming stable ion-pairs with antibiotics to protect against degradation and ensure targeted delivery [140].
(C) Hydrogels: The primary function of hydrogels is to serve as adjuvants, thereby facilitating the efficacy of antibiotics in overcoming bacterial resistance. It has been demonstrated that these metals exhibit a positive charge, which has been shown to disrupt the negatively charged cell walls of bacteria with a low toxicity for mammalian cells [141].
(D) Antimicrobial nanoparticles: The delivery of antimicrobial agents to the site of infection can be facilitated by the use of nanoparticles, which allow for targeted delivery directly to the site of infection, including drug-resistant bacterial biofilms [142]. Their small size not only facilitates the transfer of antibiotics into cells, but also enables the destruction of bacteria through the inhibition of DNA and enzymes synthesis, the inactivation of proteins, and the induction of reactive oxygen species (ROS). Recent studies have shown that combining these with various nanoparticles composed of metals such as silver, zinc, copper and iron is effective against most bacterial mechanisms of antibiotic resistance [143]. Silver nanoparticles have been identified as a promising solution for combating *P. aeruginosa* infections due to their ability to disrupt bacterial membranes and biofilms [144].
(E) Phage therapy as antibiotic alternative: The employment of bacterial viruses, known as phages, for the treatment of bacterial infections, a process referred to as phage therapy, was first documented in the early years of the 20th century [145]. This therapy has been demonstrated to be efficacious and advantageous due to its bactericidal mode of action, low intrinsic toxicity and lower risks of microbial resistance. However, despite its documented efficacy in several clinical cases, its general approval is still pending and research is ongoing to ascertain its safety and efficacy for broader therapeutic use [146].

## 5. Preventive Measures

**Figure 3:**
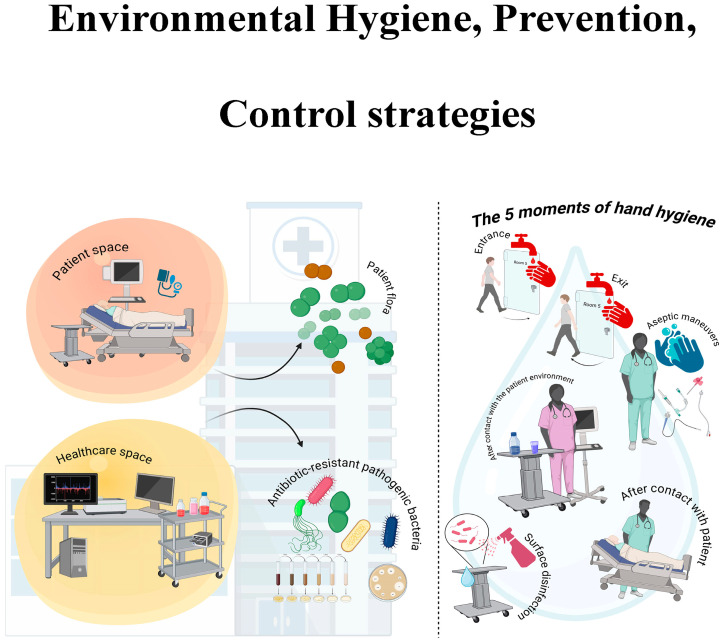
Environmental Hygiene, Prevention and Control. Figure created using BioRender.

Ensuring patient safety is of paramount importance in the healthcare sector [147]. Nevertheless, there are a multitude of medical practices that have the potential to compromise patient health. Evidence suggests that such practices have a detrimental effect on patient health, often compromising or delaying recovery [148]. This problem affects both high-income countries and low- and middle countries even if priorities may differ. These infections, frequently caused by antibiotic-resistant organisms and transmitted indirectly through contact with contaminated staff or equipment, compromise the effectiveness of treatments [4]. Strict hygiene measures, appropriate antibiotic use and the use of protective equipment are fundamental elements in combating this problem [149]. The incorporation of error prevention into design, in conjunction with well-structured tasks and processes, serves to mitigate the likelihood of system errors [150] (Figure 3).

In a variety of healthcare contexts, the objective is to prevent the transmission of pathogens from the “patient zone” to the broader “healthcare zone” by distinguishing and protecting these areas. Strategies to prevent and control environmental hygiene aim to reduce HAIs by cleaning/disinfecting surfaces, water, and air; managing waste and equipment properly; and implementing proper infrastructure and staff training. Hand hygiene is crucial for healthcare workers to prevent the transmission of harmful microorganisms and reduce infections. They should practise hand hygiene at these pivotal points: before touching a patient, before a clean/aseptic procedure, after risk of exposure to body fluids, after touching a patient, and after touching patient surroundings.

In order to facilitate continuous improvement, it is imperative that immediate access to past error data is permitted. The establishment of a culture of safety is contingent upon an environment that is both open and transparent, with involvement at all levels of the organisation. The establishment of an efficient reporting system is considered to be a fundamental component for healthcare organisations [151]. It is responsible for the collection of experiences and data (for example, adverse events and incidents) and the provision of feedback from professionals. Furthermore, “second victims” of adverse events are healthcare professionals. It is possible that they may experience a decline in confidence with regard to their clinical skills and knowledge [152]. Furthermore, they may find themselves confronted with a diminution in job satisfaction, which could result in their contemplation of departing from the healthcare profession. This, in turn, has the potential to have a negative impact on patient safety. There are pragmatic measures that countries around the world can take to protect their healthcare systems from the threat of AMR. First, it is essential to implement stronger measures to facilitate infection monitoring and prevention at the national and hospital levels [22]. At the same time, access to vaccines and sanitation services should be expanded. Secondly, it is essential to optimise the use of antibiotics not used to treat human diseases, such as in food and animal production, by adopting a One Health approach and recognising the interconnection between human and animal health [153]. In this context, antimicrobial treatments require particular attention. This means ensuring sufficient availability of life-saving antibiotics and minimising consumption were unnecessary. These measures must be implemented in accordance with the Global Action Plan established by the World Health Organization (WHO) and the AWaRE guidelines [154]. Furthermore, it is imperative to augment funding at all stages of the process of developing new antimicrobials, with a particular focus on high-priority bacterial strains (*K. pneumoniae*, *P. aeruginosa*, etc.). Moreover, innovative market solutions should be employed to facilitate access to these antimicrobials especially in low- and middle-income countries [155]. Active surveillance strategies are vital to preventing the spread of HAI, but are often limited by their labour-intensive nature, which can be time-consuming and susceptible to errors. Recent research suggests that there is considerable potential for the utilisation of artificial intelligence (AI) to enhance the efficacy of HAIs prevention through the application of sophisticated predictive capabilities [156]. Earlier research has demonstrated the efficacy of an ECDC-based algorithm (CRI3-CVC) in identifying CVC-BSI cases, exhibiting comparable performance to manual methodologies, while reducing the workload for healthcare personnel [157]. Furthermore, research is currently underway to develop a machine learning-based predictive model to differentiate between hospitalised patients at low risk and those at high risk of BSI [158]. The development of these predictive models of BSI is also fundamental in the context of AMR, which is primarily caused by the inappropriate use of antimicrobials. The employment of these models has the potential to curtail unwarranted exposure to antibiotics in instances where the likelihood of developing a bloodstream infection (BSI) is negligible [159]. Two distinct artificial intelligence models are currently under development each tailored for a specific purpose. The first predicts how susceptible microorganisms are to different antibiotics. The second creates new antimicrobial peptides capable of attacking cell membranes and against which microorganisms develop a low degree of resistance [160].

## 6. Conclusions

Hospital-acquired infections present a paradox: firstly, that a place designed for patient healing and treatment becomes a source of risk for the patients themselves, who contract infections while receiving care; and secondly, that the very purpose of the hospital, to heal and treat patients, is rendered null and void. These infections, categorised as HAIs, pose a significant challenge, often resulting in protracted hospitalisation, elevated risk of disability and the spread of antibiotic resistance. The most common isolated microorganism was *P. aeruginosa* in ICU-acquired pneumonia, coagulase-negative staphylococci in ICU-acquired BSIs and *Escherichia coli* in ICU-acquired UTIs [114]. These and other pathogens have the potential to rapidly lead to the deterioration of patients’ underlying clinical conditions. A strategy that has been adopted by hospitals in an effort to combat resistance involves the restricted use of so-called “last resort” drugs. The underlying rationale for this approach is to regulate access to broad-spectrum drugs. It has been demonstrated in previous studies that the limitation of antibiotic agents such as imipenem has been found to be associated with a decrease in the development of resistance, particularly in relation to certain pathogens, including *Pseudomonas aeruginosa* and *A. baumannii*. In the context of poorer countries, it has been documented that fewer than 7% of individuals afflicted with severe drug-resistant infections receive the necessary antibiotics, a situation which has been shown to both exacerbate morbidity and mortality rates and to contribute to the development of AMR. The utilisation of alternative low-efficiency antibiotics is likely to result in a protracted duration of antibiotic usage. This phenomenon has been associated with an escalation in resistant bacteria. The administration of appropriate treatment would result in the eradication of drug-resistant bacteria, thereby mitigating their propagation. Errors in healthcare are prevalent, as evidenced by the well-known adage, “to err is human”. A report published in 1999 in the United States revealed that the number of fatalities resulting from medical errors exceeds the number of fatalities caused by motor vehicle accidents, breast cancer and AIDS. The report posits that the crux of the issue does not lie in the presence of incompetent individuals within the healthcare sector, but rather in the inadequacy of the underlying systems that they are operating within. In many countries worldwide, support for patient safety practices has been fostered through the establishment of international/national plans, networks and organisations. One of the most significant resolutions is that pertaining to patient safety (WHA72.6), which was adopted by 194 countries participating in the 72nd World Health Assembly, held in Geneva. This resolution formally acknowledged that patient safety should be considered a matter of global significance for health, thus prompting the formulation of the WHO Global Action Plan for Patient Safety for the years 2021 through to 2030. The Plan’s objective is to enhance the safety of care through the dissemination of information and experience, and the implementation of good practices. In order to surmount this challenge, there is a necessity for collaboration between healthcare professionals and patients. Transparency is of paramount importance: members of the public must be cognisant of healthcare organisations’ endeavours to avert harm. It is imperative to reverse the prevailing perception that hospitals are more hazardous than they are. It is incumbent upon all healthcare organisations to ensure that their staff receive training in specific safety issues.

## Figures and Tables

**Table 1:** Common types of medical errors and associated complications.

Medical Errors	Examples	Associated Complications
Healthcare-Associated Infections	Pneumonia, surgical site, urinary, gastro-intestinal and bloodstream infections.	Longer hospital stays, disability, drug resistance and cost.
Surgical errors	Wrong-site surgery, wrong-patient surgery and instruments left inside the patient.	Bleeding, infection, tissue/organ damage, death.
Diagnostic errors	The misinterpretation of physical, laboratory or radiologic findings	Heart failure, acute kidney failure, sepsis, pneumonia, respiratory failure and hypoxaemia.
Unsafe Injections	Improper use of syringes and needles or multiple injections using a single needle and syringe	Transmission of bloodborne pathogens like HCV, HBV and HIV, and abscess formation.
Radiation Errors	Incorrect dose of radiation, wrong site, incorrect energy and geometric misses.	Fatal injuries can be life-threatening if they affect vital organs like the spinal cord, heart, lungs, or brain.
Sepsis	Sepsis most commonly originates in the lungs, abdomen and urinary tract.	Multiple organ failure and decreased cognitive functioning
Unsafe Transfusion	Unsafe donors, poor testing procedures, poorly trained staff and unnecessary transfusions.	Adverse transfusion reactions (i.e., acute immune haemolytic reaction) and transfusion-transmissible infections.
Medication Errors	Unauthorised medication, incorrect administration, administering to the wrong patient, extra dose, wrong rate, or failure to communicate important findings.	Rashes, itching, skin disfigurement, organ damage, respiratory problems.
